# Methamphetamine Exposure Induces Neuronal Programmed Necrosis by Permeabilizing Mitochondria via the RIPK1–RIPK3–MLKL Axis

**DOI:** 10.3390/toxics13090736

**Published:** 2025-08-30

**Authors:** Peng Zhou, Jiankang Xuan, Weixiao Xu, Di An, Sining Meng, Hongchao Zhang, Miaoyang Hu, Wanqingyang Hui, Yifei Wang, Jie Cheng, Jianping Xiong, Jun Wang, Xufeng Chen

**Affiliations:** 1 Department of Emergency Medicine, the First Affiliated Hospital of Nanjing Medical University, 300 Guangzhou Road, Nanjing 210029, China; pengzhou@stu.njmu.edu.cn (P.Z.); wangyifeivia@outlook.com (Y.W.); 2 Departoment of Emergency Medicine, Affiliated Peple’s Hospital of Jiangsu University, Zhenjiang 212002, China; 3 Center for Global Health, School of Public Health, Nanjing Medical University, Nanjing 211166, China; 4 Department of Hygienic Analysis and Detection, School of Public Health, Nanjing Medical University, 101 Longmian Avenue, Nanjing 211166, China; 5 The Key Lab of Modern Toxicology (NJMU), Ministry of Education, School of Public Health, Nanjing Medical University, 818 Tianyuan East Road, Nanjing 211166, China

**Keywords:** Meth, Mitochondrial dysfunction, neuronal necroptosis, RIPK1–RIPK3–MLKL axis

## Abstract

Methamphetamine (Meth), a psychostimulant drug of the amphetamine type, is widely abused and highly neurotoxic. Meth exposure leads to neuronal necroptosis, and the mitochondrial dysfunction may be involved. However, the underlying mechanisms remain poorly understood. Here, we found that Meth significantly elicited the formation of the RIPK1–RIPK3–MLKL necrosome complex. Intriguingly, the activated MLKL (p-MLKL) translocated to the mitochondrial membrane and displayed pore-forming activity, manifesting as the penetration of MLKL in the cell membranes of the mitochondria, which caused decreased mitochondrial membrane potential, ATP generation, and mitochondrial DNA (mtDNA) and increased mitochondrial ROS (mtROS) generation, which finalized neuronal necroptosis. Notably, MLKL activation and translocation seem to depend on the RIPK1–RIPK3 axis since these adverse effects can be substantially ameliorated by disruption of the necrosome complex formation by the necroptotic inhibitor 1 (Nec-1), which also markedly impeded the MLKL mitochondrial membrane translocation. Finally, to delineate the effects of pore formation-associated ROS generation, specific blockage of mtROS retarded the Meth-induced neuronal necroptosis. In conclusion, our study reveals for the first time that MLKL mitochondrial membrane translocation may be involved in Meth-induced neuronal necroptosis. Therefore, impeding MLKL translocation might provide a novel therapeutic strategy for Meth-induced neurotoxicity.

## 1. Introduction

Methamphetamine (Meth), an amphetamine-type psychostimulant abused globally, presents a significant public health concern [1]. Long-term or high-dose Meth use contributes to severe damage to health, including neurotoxicity, cardiovascular toxicity, and hepatotoxicity [2,3,4]. Notably, the central nervous system (CNS) is one of the significant toxic targets for Meth abuse, involving multifaceted mechanisms such as dopamine depletion, oxidative stress, neuroinflammation, excitotoxicity, and the apoptotic and autophagic pathways [5,6]. Accumulating evidence suggests that Meth can induce necrosis of neurons [7,8], which has been associated with ischemia reperfusion injury [9], Alzheimer’s disease (AD), and amyotrophic lateral sclerosis (ALS) [10,11,12,13,14]. Thus, targeting the necroptotic signaling pathway may offer a promising strategy for Meth intervention.

Necrosis was previously considered a form of non-programmed cell death, distinct from apoptosis regulated by caspase cascade reactions [15]. However, accumulating studies indicated that necrosis, like apoptosis, can be executed by regulated mechanisms. Now, an extensive network of genes regulates necroptosis, a specialized form of programmed necrosis [16], which is closely associated with Meth-induced neurotoxicity. Research has shown that cortical neurons from rats exhibit dose- and time-dependent declines in viability upon Meth treatment, notably inducing significant necroptosis [8]. Meanwhile, its exposure increases the expression of RIPK3 and MLKL, which can be partially inhibited by the RIPK1 inhibitor Necrostatin-1 (Nec-1). Furthermore, Meth exposure can induce necroptosis in both human and mouse striatal neurons [7] indicating the prominent roles of the necroptotic signaling pathway in Meth-induced neurotoxicity.

During the process of necroptosis, receptor-interacting protein kinase 1 (RIPK1) plays a crucial initiating role. It interacts with receptor-interacting protein kinase 3 (RIPK3) through its kinase domain, leading to the phosphorylation of RIPK3, then promotes the formation of the RIPK1–RIPK3 protein complex, serving as a platform to stimulate the phosphorylation of mixed-lineage kinase domain-like protein (MLKL). The phosphorylated MLKL (p-MLKL) undergoes a conformational change, promoting its oligomerization, with a high affinity for lipid bilayers and a tendency to insert into the membrane to form pores, causing membrane rupture [17]. On the basis of the molecular mechanism, the application of treatment targeting MLKL extends to potential neuroprotective strategies for brain-adverse outcomes, such as temporal lobe epilepsy, where necroptosis inhibition with Nec-1s attenuated astrogliosis, neuronal loss, and histopathological alterations [18]. However, whether Meth induces these adverse effects on mitochondria, an organelle with membranal structural similarities with the plasma membranes, to date is unknown.

Historically, mitochondria have been regarded as key effectors of necroptotic signals, as mitochondrial swelling and damage have been observed in tumor necrosis factor (TNF)-induced RIPK3-mediated cell death [19]. The N-terminal four-helix bundle of MLKL oligomerizes to breach the plasma membrane, influencing necroptosis in a manner similar to gasdermins, and preferentially interacts with cardiolipin, suggesting a functional connection with mitochondria [20]. Neurons heavily rely on mitochondria due to their complex morphology and high energy demands [21,22,23,24]. Mitochondrial damage has been implicated in a range of pathological processes, including apoptosis, reactive oxygen species (ROS) production, lipid metabolic dysregulation, and defects in intracellular energy production. For instance, norovirus acquires pore-forming domains similar to those of MLKL to impair mitochondrial function and promote viral egress; under high-glucose conditions, RIPK1 and MLKL form oligomers in an activated phosphorylated state within mitochondria [25]. For Meth-related mitochondrial injury, available reports indicated that Meth exposure leads to mitochondrial dynamic disruption and neurodegeneration [26,27]. Additionally, Meth impairs the activity of mitochondrial complex II-III in the rat striatum [28] and induces the release of cytochrome c (Cytc) in PC12 cells and human neuroblastoma SH-SY5Y cells via the activation of mitochondrial apoptotic pathways [29]. Apart from the striatum, studies have also shown that Meth exposure affects mitochondrial complex I activity in the hippocampus [30], leading to energy metabolism disorders. Similarly, Meth has been found to disrupt mitochondrial complex IV function in the prefrontal cortex [31], contributing to oxidative stress and neuronal damage. These findings prompt us to investigate whether MLKL is involved in Meth-induced mitochondrial damage, leading to necroptosis.

In macrophages and fibroblasts, mitochondria-derived reactive oxygen species (mtROS) contribute to necroptosis via multiple mechanisms, including promoting RIPK1 autophosphorylation and necroptotic signaling. RIPK3 interacts with MLKL-dependent mitochondrial pyruvate dehydrogenase complexes, stimulating oxidative phosphorylation and mtROS production to promote necroptosis [32]. Moreover, B-cell lymphoma 2 (BCL2) proteins are associated with necroptosis, as the absence of BCL2-associated X protein (BAX) and BCL2-antagonist/killer (BAK) impairs Tumor Necrosis Factor (TNF)- or glucocorticoid-induced RIPK1- and RIPK3-mediated cell death [33]. These findings underscore the vital role of RIPK1–RIPK3–MLKL necroptotic signaling in necroptosis via mitochondria, implying that MLKL inhibition may offer a promising therapeutic avenue to mitigate the neurodegenerative consequences of Meth exposure.

Noteworthily, previous studies have mainly focused on the role of MLKL in the plasma membrane, given the structural similarities between the plasma and mitochondrial membranes. In the present study, MLKL was selected as a key investigative target based on its well-characterized function as the terminal executor of necroptosis, where it mediates membrane disruption through its pore-forming capability in the plasma membrane, particularly in mitochondrial membranes in the context of Meth-induced neurotoxicity. Therefore, the present work extends a novel insight to existing knowledge on Meth’s neurotoxic mechanisms, by the translocation of MLKL to the mitochondrial membrane, contributing to mitochondrial dysfunction and neuronal necroptosis. Additionally, the pharmacological inhibition of RIPK1 and mtROS was exploited to verify the deteriorated effects of RIPK1–RIPK3 signaling in mitochondrial damage, and the mitochondria-derived neuronal necroptosis mediated by Meth was discussed as well.

## 2. Materials and Methods

### 2.1. Reagents

Meth (National Institutes for Food and Drug Control, China) was dissolved in sterile saline (0.9% NaCl) to generate a concentration gradient (0, 100, 300, 600, 900 μM). Necrostatin-1 (Nec-1; HY-15760, MedChemExpress, Beijing, China) was prepared as a 10 mM stock in DMSO and diluted to working concentrations (20 μM) with solvent controls containing ≤ 0.1% DMSO. MitoTEMPO (mtT; ab144644, Abcam, Boston, MA, USA) was dissolved in ultrapure water (50 mM stock) and used at 5 μM, with matched water controls. All treatments were administered after neuronal maturation (about 10 days).

### 2.2. Primary Cortical Cell Culture

The primary cortical cells were cultured as in our previous report [5]. On embryonic day 18, primary cortical neurons were dissected from embryonic brains using a microscope (OLYMPUS, Tokyo, Japan) and digested with papain and DNase (DN25, Sigma, St. Louis, MO, USA). A single neuron suspension was generated by pipetting and filtering through a 40 µm nylon strainer and then plated onto plates precoated with poly-D-lysine (P1024, Sigma, St. Louis, MO, USA). The neurons were cultured for 10 days in neurobasal medium (21003-049, Invitrogen, Carlsbad, CA, USA) with B27 (17504-044, Invitrogen, Carlsbad, CA, USA), 1% L-glutamine (25030-081, Gibco, Grand Island, NY, USA), and 0.5% penicillin/streptomycin (450-201-EL, Multicell Techs, Inc., Woonsocket, USA) in an atmosphere containing 5% CO_2_ at 37 °C. The medium was changed every 3 days. All experiments were conducted under the control of the Ethics Committee of Animal Care and Experimentation of Europe and approved by the Institutional Animals Care and Use Committee (IACUC) at Nanjing Medical University (IACUC-201902013).

### 2.3. Hoechst 33342/Propidium Iodide (PI) Dual Staining

PI-positive cells were considered dead as PI uptake indicates the loss of cell membrane integrity. For morphological analysis, neurons were incubated with PI (ST1569, Beyotime, Shanghai, China) at a concentration of 2 μg/mL in the culture medium for 15 min at 37 °C. Following incubation, unbound PI was not removed by washing to maintain staining integrity during observation. Fluorescence images were then acquired using a fluorescence microscope (Carl Zeiss LSM900, Jena, Germany) with excitation at 535 nm and emission detection at 617 nm.

### 2.4. Lactate Dehydrogenase (LDH) Detection

LDH release was quantified using a commercial LDH Cytotoxicity Assay Kit (C0019S, Beyotime, Shanghai, China) to assess cellular injury. Briefly, neurons were treated with Meth according to experimental groupings. Following treatment, culture supernatants were collected via centrifugation and transferred to a fresh 96-well plate (100 μL/well). LDH detection working solution (10 μL) was added to each supernatant-containing well, followed by incubation at 37 °C for 20 min to facilitate the enzymatic reaction. Absorbance was measured at 450 nm by a microplate reader. LDH concentrations were determined by normalizing sample absorbance values against a concurrently generated standard curve.

### 2.5. Mitochondrial Membrane Potential Detection

Neurons were washed with PBS and incubated in the JC-1 staining buffer (5×) (C2005S, Beyotime, Shanghai, China) for 15 min at 37 °C. After washing with PBS, cells were subjected to confocal laser scanning microscopy (Carl Zeiss LSM900, Jena, Germany).

### 2.6. MitoROS Production Assay

MitoROS production in neurons was assessed using the MitoSOX Red reagent (M36009, Thermo, Waltham, MA, USA). Staining with 2 μM MitoSOX reagent was performed in live primary neurons for 10 min at 37 °C. The fluorescence was examined using laser scanning microscopy (Carl Zeiss LSM900, Jena, Germany), and pictures were randomly captured for each group. The mean fluorescence intensity of these pixels was calculated using ImageJ (Version 1.53, NIH, USA).

### 2.7. Quantitative RT-PCR

Total RNA was extracted from cell lysates using TRIzol (343,903, Thermo, Waltham, MA, USA) reagent according to the manufacturer’s instructions. A NanoDrop spectrophotometer (NanoDrop 2000, Thermo, Waltham, MA, USA) was used to determine the quantified total RNA. Relative mRNA levels were detected using SYBR Green Supermix (11201ES08, Yeasen Biotech Co., Ltd., Shanghai, China). Changes in fluorescence were monitored on a LightCycler 480 (Roche). GAPDH was used as the internal control. The sequences of the primers used for RT-PCR are listed (Supplemental Table S1).

### 2.8. mtDNA Copy Number

The residual aqueous layer on the intermediate layer was removed according to the RNA isolation protocol (Accurate Biology, China), and the samples were mixed by adding 0.3 mL of anhydrous ethanol for every 1 mL of TRIzol (343,903, Thermo, Waltham, MA, USA) as initially done, with repeated inversion. The samples were then centrifuged at 2000× *g* for 5 min at 4 °C to precipitate the DNA; then, the precipitates were washed twice with 0.1 M sodium citrate (prepared in 10% alcohol) and resuspended with 75% alcohol before being centrifuged at 2000× *g* for 5 min at 4 °C [34]. The relative copy number of mitochondrial DNA was quantified using real-time quantitative PCR (LC480) and normalized to simultaneously determine nuclear DNA amplification [35]. Specifically, the amplification of mitochondrial Cytochrome b (Cytb) and ND1 (mitochondrial-encoded genes) was normalized to HK2 (a nuclear-encoded gene).

### 2.9. ATP Detection

Cellular ATP content was quantified using a commercial firefly-luciferase-based ATP assay kit (ATPlite, S0027; Beyotime, Shanghai, China) following manufacturer protocols. Briefly, cells were lysed on ice for 30 min using ice-cold lysis buffer provided with the kit. Lysates were centrifuged (12,000× *g*, 10 min, 4 °C), and supernatants were collected for analysis. Luminescence was measured using a microplate luminometer (Berthold Technologies, UK). ATP concentrations were normalized to total protein content determined by BCA assay, with results expressed as n mol ATP/mg protein. Cells maintained in Meth-free medium served as negative controls, with six biological replicates per experimental group.

### 2.10. Western Blot Assay

As described previously [5], total proteins were extracted from cortical neurons in ice-cold Radio Immunoprecipitation Assay Lysis buffer (R0278, Sigma, St. Louis, MO, USA) containing protease inhibitor and phosphatase inhibitor cocktails (1:500, Sigma, St. Louis, MO, USA), then the protein concentrations were determined by bicinchoninic acid (BCA) protein assay (Thermo, Waltham, MA, USA). Afterward, electrophoresis separated them on 8–15% SDS-PAGE gels (Bio-Rad, California, USA) and transferred them to polyvinylidene difluoride membranes (Millipore Corporation, Massachusetts, USA). Then, the membranes were blocked with 5% non-fat milk or 1% bovine serum albumin for 1 h at room temperature and incubated overnight at 4 °C with the primary antibody in Tris-buffered saline containing 0.1% Tween and 5% non-fat milk or 1% bovine serum albumin. The following primary antibodies were used: TNF-α (17590-1-AP, Proteintech, Chicago, USA); RIPK1 (3493, Cell Signaling Technology, Danvers, MA, USA); p-RIPK3 (AF7443, Affbiotech, Wuhan, China); RIPK3 (sc-374639, Santa Cruz, CA, USA); MLKL (ab184718, Abcam, Boston, USA); p-MLKL (ab196436, Abcam, Boston, USA); β-actin (sc-376421, Santa Cruz, CA, USA). Protein bands were detected by enhanced chemiluminescence (Thermo, Waltham, MA, USA). The optical density of a band represents its protein content (ImageJ, USA). β-actin was used as a loading control.

### 2.11. Immunofluorescence Analysis

The neurons were incubated with the diluted MitoTracker (Invitrogen, Carlsbad, CA, USA) at 37 °C for 15 min, after washing, fixed with ice Methanol for 15 min, and permeabilized with 0.1% Triton X-100. Non-specific binding was blocked by incubation in 10% goat serum at room temperature for 1 h. Samples were then incubated with the following primary antibodies: p-MLKL (ab196436, Abcam, Boston, MA, USA); MLKL (ab184718, Abcam, Boston, MA, USA); RIPK3 (sc-374639, Santa Cruz, CA, USA) overnight at 4 °C, and then washed with PBS. Signals were developed with Alexa fluorescence antibodies (P11047/L21416, Invitrogen, USA). Finally, the cells were stained with DAPI dihydrochloride. Confocal microscopy analysis was performed using an LSM microscope (Carl Zeiss LSM900, Jena, Germany).

### 2.12. Quantitative Analysis

Data are presented as mean ± standard deviation. Data were analyzed using the Student’s *t*-test or ANOVA (Dunnett’s test). Analyses were performed using GraphPad Prism 9 software. All statistical tests were two-tailed, and a *p*-value < 0.05 was considered statistically significant.

## 3. Results

### 3.1. Methamphetamine Induces Necroptosis in Primary Neurons

**Figure 1:**
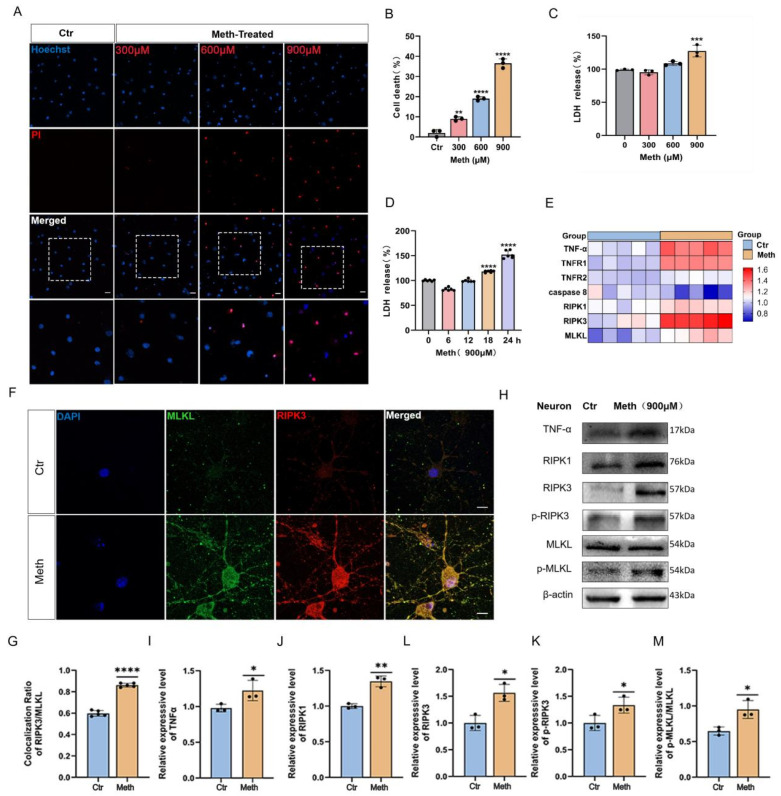
Neuronal necroptosis induced by Meth. (**A**) The primary neurons were exposed to 0, 300, 600, and 900 μM Meth for 24 h, and neuronal death was assessed by PI staining. Scale bars, 20 μm. (**B**) quantitative analysis of PI staining in neurons (50 neurons/group; n = 3). (**C**,**D**) The primary neurons were exposed to 0, 300, 600, and 900 μM Meth for 24 h or 0, 6 h, 12 h, 18 h, and 24 h, and levels of LDH were determined. (**E**) The primary cortical neurons were treated with Meth (900 μM) for 24 h. TNF-α, TNFR1, TNFR2, RIPK1, RIPK3, and MLKL mRNA levels were detected by qRT-PCR normalized to untreated neurons. (**F**) Representative images of immunofluorescence staining of RIPK3 (red), MLKL (green), and DAPI (blue) after 900 μM Meth (24 h). Scale bars, 10 μm. (**G**) Quantification of RIPK3/MLKL co-localization (50 neurons/group; n = 3). (**H**) The protein expression of TNF-α, RIPK1, RIPK3, p-RIPK3, and p-MLKL after 900 μM Meth (24 h). (**I**–**M**) Quantitative analysis in neurons of (**I**) TNF-α, (**J**) RIPK1, (**K**) RIPK3, (**L**) p-RIPK3, (**M**) p-MLKL (gray value analysis; n = 3). β-actin was used as a loading control. Data are expressed as the mean ± SD. Experiments were repeated at least three times. * *p* < 0.05, ** *p* < 0.01, *** *p* < 0.001, **** *p* < 0.0001 compared to the control group.

To assay the Meth-induced necroptosis in primary neurons, PI staining and LDH assay were applied. As expected, Meth treatment elicited a striking plasma membrane rupture and cellular content leakage. To address the specific toxic effects of Meth, the dose- and time-dependent responses were investigated. It showed that, after Meth exposure, the number of PI-positive cells significantly increased with the peak response at 900 μM. In parallel, this dosage elicited a significant increase in LDH release (Figure 1A,B). Previous studies have demonstrated that Meth induces neuroglial cell death (EC_50_ = 1 mM) and that binge dosage of 250 mg to 1 g of Meth results in a range of 164–776 μM of Meth concentration in the brain [36,37]. Furthermore, the LDH assay showed that Meth exposure resulted in LDH release in a dose-dependent manner, with peak response at the concentration of 900 μM Meth (Figure 1C). Given these results, 900 μM Meth was chosen in the subsequent experiments. Meanwhile, the time course reaction (6 h, 12 h, 18 h, and 24 h) of Meth was assessed, and it showed that LDH release increased over 18 h, with a maximal effect at 24 h (Figure 1D). To further support that Meth causes neuronal death via necroptosis, the mRNA changes associated with necroptosis were examined. As depicted in Figure 1E, Meth significantly upregulated the expression of TNF-α, TNFR1, TNFR2, RIPK1, RIPK3, and MLKL, which validated our assumption (Supplementary Figure S1).

MLKL, a terminal-known obligate effector in necroptosis, plays a key role in the formation of transmembrane pores and the leakage of cell contents [38]. To address whether MLKL is involved in Meth-induced neuronal necroptosis, the overlapping immunofluorescence staining of RIPK3 and MLKL was examined. It showed a pronounced increase in the colocalization between RIPK3 and MLKL (Figure 1F). Additionally, the levels of proteins associated with necroptosis significantly increased, including TNF-α, RIPK1, RIPK3, p-RIPK3, and p-MLKL (Figure 1G,H), suggesting that Meth triggers necroptosis in primary cortical neurons.

### 3.2. Methamphetamine Induces Mitochondrial Dysfunction

**Figure 2:**
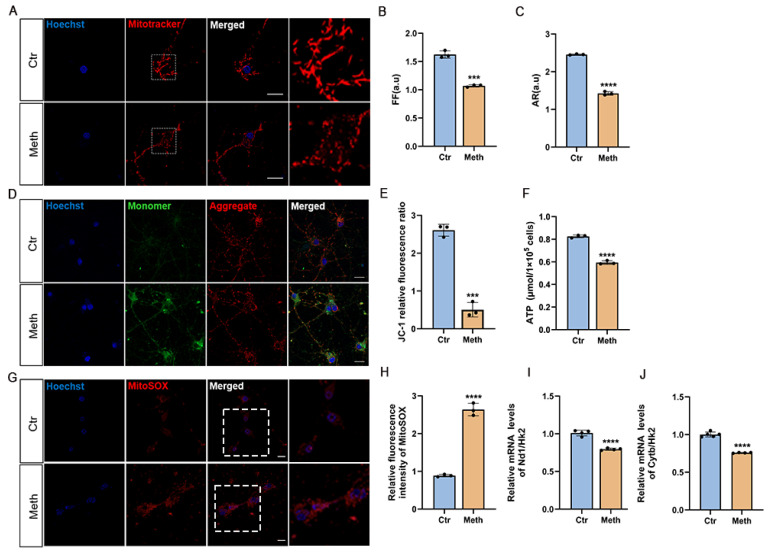
Meth induces mitochondrial defects in primary cortical neurons. (**A**) Representative mitochondrial tracker images after 900 μM Meth (24 h) or vehicle control. Scale bars, 10 μm. (**B**,**C**) Quantitative analysis of mitochondrial (**B**) form factor and (**C**) aspect ratio (30 mitochondria/neuron, 15 neurons/group; n = 3). (**D**) Images of Meth-treated neurons (900 μM, 24 h) stained with mitochondrial membrane potential (JC-1) (red indicates normal membrane potential, and green indicates low membrane potential). Scale bars, 10 μm. (**E**) Quantification of JC-1 red/green ratio (50 neurons/group; n = 3). (**F**) ATP levels after 900 μM Meth (24 h) treatment. (**G**,**H**) mtROS (MitoSOX) detection: (**G**) representative images. Scale bars, 10 μm; (**H**) quantification (50 neurons/group; n = 3). (**I**,**J**) mtDNA copy number in neurons: (**I**) experimental scheme; (**J**) quantification (n = 3). Data are expressed as the mean ± SD. Experiments were repeated at least three times. The images shown are representative of a single experiment. *** *p* < 0.001, **** *p* < 0.0001 compared to control group.

To investigate how Meth induces neuronal necroptosis, mitochondrial integrity and membrane potential were examined. Using the mitochondrial transmembrane potential-sensitive probe JC-1 and the mitochondrial superoxide indicator MitoSOX, striking alterations in mitochondrial morphology were observed via confocal laser scanning microscopy (LSM900). Specifically, Meth treatment induced a dramatic shift from predominantly rod-shaped/linear mitochondria in control neurons to fragmented, rounded organelles (Figure 2A). Quantitative analysis revealed a significant reduction in both mitochondrial morphology factor (1.625 ± 0.06 in controls vs. 1.06 ± 0.02 after Meth treatment; ~1.53-fold decrease) and aspect ratio (2.45 ± 0.04 vs. 1.42 ± 0.06; ~1.73-fold decrease), with these differences being statistically significant (Figure 2B,C). This marked transition from rod-shaped/linear to rounded mitochondria indicates profound mitochondrial fragmentation. To assess the mitochondrial membrane potential (MMP), the specific dye JC-1 was used. In healthy mitochondria, JC-1 forms red aggregates, while in a depolarized state, it exists as green cytoplasmic monomers. Our results showed a noticeable reduction in JC-1 aggregates paralleled with increased green monomers in neurons after treatment of Meth (Figure 2D,E), reflecting the reduction in the MMP. Due to the direct impacts of MMP on ATP generation, we then moved to investigate whether Meth affects ATP levels; as shown in Figure 2F, Meth treatment elicited a pronounced decline in ATP levels. Correspondingly, the mitochondria-derived ROS was substantially elevated using the mitochondria-targeted superoxide indicator MitoSOX (Figure 2G,H), and a significant decrease in mtDNA copy number was also validated (Figure 2I,J). These results, taken together, suggest that mitochondria served as the critical target organelles for Meth.

### 3.3. The RIPK1–RIPK3 Axis Is Involved in Meth-Induced Mitochondrial Damage

**Figure 3:**
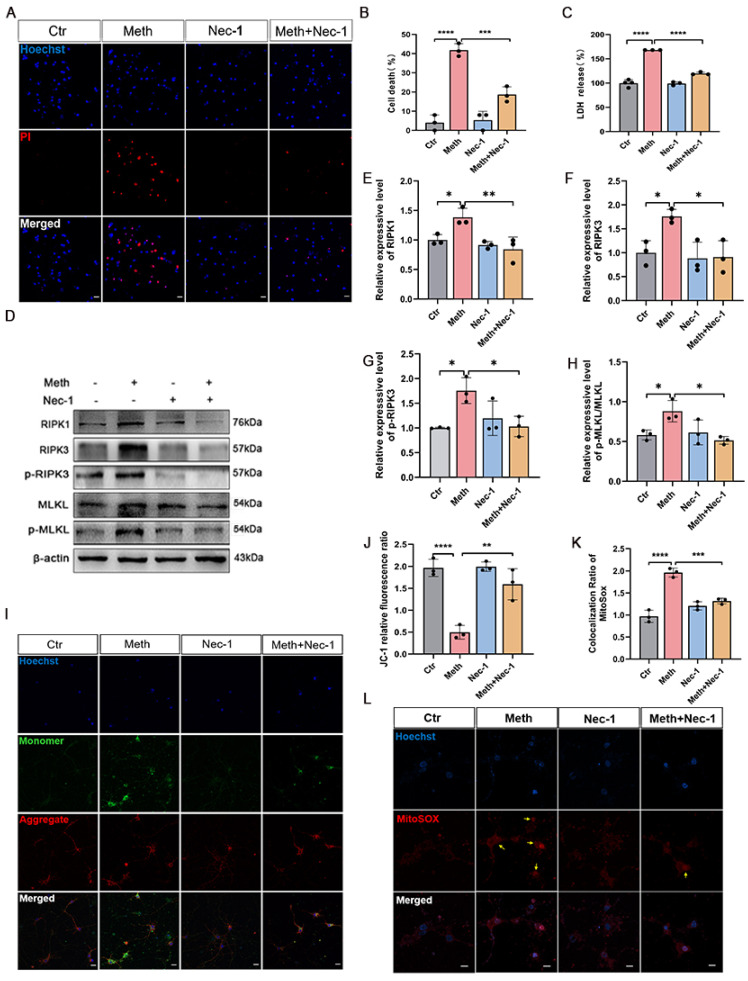
Effects of RIPK1–RIPK3 axis in Meth-induced neuronal necroptosis. (**A**) Representative PI staining images of neurons treated with 900 μM Meth (24 h) or vehicle control (containing 0.1% DMSO). Scale bars, 20 μm. (**B**) Quantitative analysis of PI-positive cells (50 neurons/group; n = 3). (**C**) Levels of LDH release after 900 μM Meth (24 h) treatment. (**D**–**H**) Western blot and quantification of protein levels (gray value analysis; n = 3) of RIPK1, RIPK3, p-RIPK3, and p-MLKL expression in neurons exposed to 900 μM Meth (24 h). (**I**–**L**) Nec-1 (20 μM) was administered 2 h before JC-1 and mitochondrial ROS (MitoSOX) staining after 900 μM Meth (24 h). Scale bars, 10 μm. Yellow arrows indicate mitochondrial recruitment of ROS (50 neurons quantified/group; n = 3). Scale bars, 10 μm. Data are expressed as the mean ± SD. Experiments were repeated at least three times. * *p* < 0.05, ** *p* < 0.01, *** *p* < 0.001, **** *p* < 0.0001 compared to the control group.

To gain more mechanistic insight into the neuronal necroptosis of Meth, the roles of the RIPK1–RIPK3 axis were explored. Therefore, Nec-1, one inhibitor for blockage of RIPK1–RIPK3 interaction, was applied (Figure 3). Moreover, LDH release and PI staining after Meth exposure were dramatically decreased in the presence of Nec-1 (Figure 3A–C). Moreover, Nec-1 treatment ameliorated the Meth-induced necroptosis since the expression of RIPK1, RIPK3, p-RIPK3, and p-MLKL proteins was significantly reduced (Figure 3D–H). Nonetheless, Nec-1 treatment significantly ameliorated Meth-induced MMP decrease (Figure 3I,J) and mitochondrial ROS (Figure 3K,L) enhancement in neurons. Altogether, these pieces of evidence highlight the critical roles of the RIPK1–RIPK3 axis in Meth-induced neuronal necroptosis.

### 3.4. MLKL Activation and Mitochondrial Membrane Translocation After Meth Treatment

**Figure 4:**
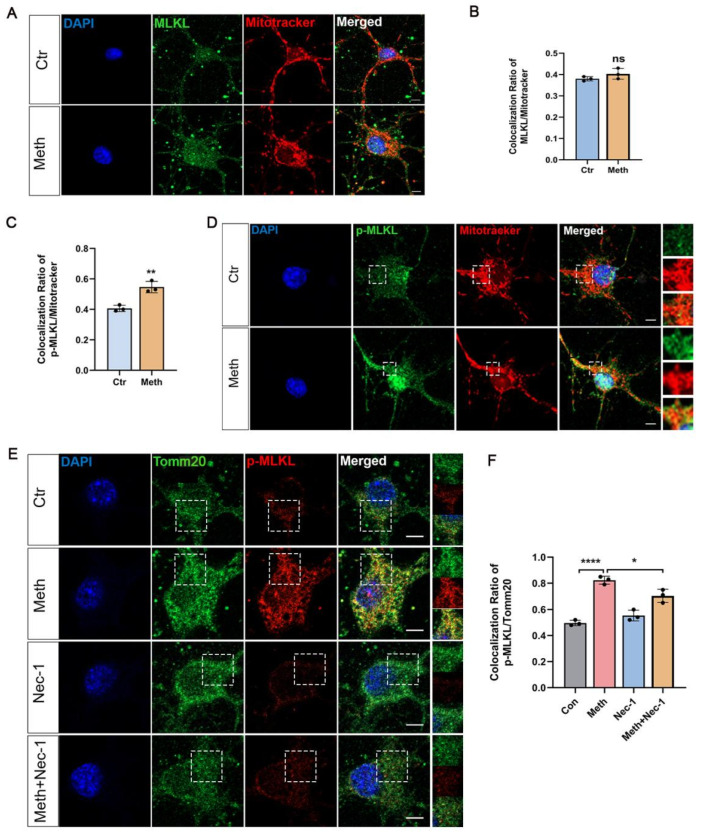
p-MLKL translocated to mitochondrial membrane driven by Meth. (**A**) Immunofluorescence staining of Mitotracker (red), MLKL (green), and DAPI (blue) in neurons treated with 900 μM Meth (24 h) or vehicle control (containing 0.1% DMSO). Scale bars, 10 μm. (**B**) Quantitative analysis of mitochondrial MLKL translocation (50 neurons/group; n = 3). (**C**) Quantitative analysis of p-MLKL fluorescence intensity (50 neurons/group; n = 3). (**D**) Images of immunofluorescence staining of Mitotracker (red), p-MLKL (green), and DAPI (blue) after 900 μM Meth (24 h). Scale bars, 10 μm. (**E**) With the pre-treatment with Nec-1 (20 μm) for 2 h followed by 900 μM Meth (24 h), images of p-MLKL (red), Tom20 (green), and DAPI (blue). Scale bars, 10 μm. (**F**) Quantitative analysis of mitochondrial p-MLKL (50 neurons/group; n = 3). Data are expressed as the mean ± SD. Experiments were repeated at least three times. * *p* < 0.05,** *p* < 0.01, **** *p* < 0.0001 compared to control group.

Having investigated the combination of the critical roles of the RIPK1–RIPK3 axis in neuronal necroptosis and the MMP decrease in Meth-induced neuronal cells, we then moved to the downstream effector protein MLKL, which is known for forming pores in the plasma membrane which impairs membrane integrity [17]. Surprisingly, after the Meth challenge, the activated MLKL (p-MLKL) rather than MLKL displayed an apparent colocalization between p-MLKL and Mitotracker (Figure 4A–D). To confirm the precise sites of the p-MLKL translocated, the mitochondrial outer membrane protein marker Tomm20 was detected for the evaluation of overlapping between p-MLKL and Tomm20. This showed that Meth exposure facilitated the colocalization of p-MLKL with Tomm20, an effect which was impeded by the administration of Nec-1 (Figure 4E,F), implying that the activated MLKL mediated by the RIPK1–RIPK3 axis exerts a pivotal impact on mitochondrial membrane penetration induced by Meth.

### 3.5. Mitochondrial ROS Amplifies Neuronal Necroptosis Induced by Meth

**Figure 5:**
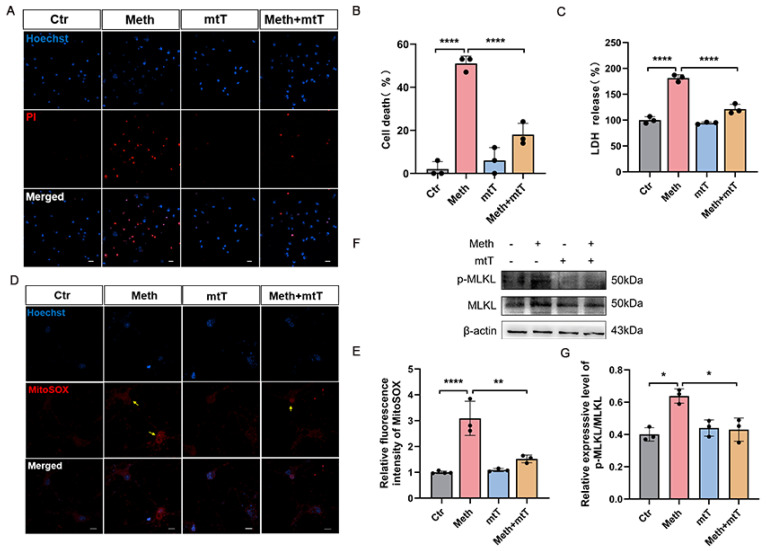
Meth-induced mtROS facilitated neuronal necroptosis. (**A**) MitoTEMPO (5 μM) treatment 2 h before Meth exposure (900 μM, 24 h), and the PI staining which was detected. Scale bars, 20 μm. (**B**) Quantitative analysis of PI-positive cells (50 neurons per experiment, n = 3). (**C**) Quantitative analysis of LDH release after Meth treatment (900 μM, 24 h; n = 3). (**D**,**E**) Images of cells stained with mitochondrial ROS (MitoSOX). Yellow arrows indicate mitochondrial recruitment of ROS. 50 neurons quantified per group (n = 3). Scale bars, 10 μm. (**F**) Western blot and quantitative analyses of the p-MLKL expression in neurons. (**G**) Quantification of p-MLKL levels (gray value analysis, n = 3). Data are expressed as the mean ± SD. Experiments were repeated at least three times. * *p* < 0.05, ** *p* < 0.01, **** *p* < 0.0001.

Since the aforementioned Meth induced the penetration of the activated MLKL in the mitochondrial membrane, we then assessed the association between the impairment of the membranal integrity and ROS generation. Previous research demonstrated that mitochondrial ROS contributes to initiating neuronal necroptosis, leading to the activation of necrosome formation. To investigate whether the Meth-induced neuronal necroptosis ascribes to the mitochondrial ROS, MitoTEMPO, a mitochondrial ROS scavenger, was applied. As expected, Meth treatment resulted in an increase in PI-positive cells (Figure 5A,B), LDH release (Figure 5C), p-MLKL protein expression (Figure 5F,G), and mitochondrial ROS levels (Figure 5D,E), whereas application of MitoTEMPO (mtT) remarkably retarded these detrimental effects, pointing out that mtROS plays key roles in activating MLKL and neuronal necroptosis, reflecting a positive feedback loop of p-MLKL penetration and ROS generation.

## 4. Discussion

In the present study, we first demonstrated that the RIPK1–RIPK3–MLKL axis is involved in Meth-induced neuronal necroptosis via an unrecognized MLKL translocation to the mitochondrial membrane which penetrated into the membrane of the mitochondria, which led to mtROS increase and decreases in MMP, ATP production, and mtDNA in neuronal cells. Thus, targeting MLKL might provide a novel intervention strategy for Meth-induced neuronal death.

The nervous system is one of the most critical targets of Meth. Meth abusers are at increased risk of developing memory impairment, depression, Parkinson’s disease, Alzheimer’s disease, and other neuropsychiatric and cognitive disorders associated with neuronal damage [39,40]. Of note, neuronal death is one of the most common characteristics of Meth-induced toxicity [41]. Previous studies have shown that mitochondrial dysfunction is involved in Meth-induced neurotoxicity [42,43], releasing apoptotic proteins in the intramembrane space [44]. Recently, more progress has been made in the mitochondrial damage induced by Meth, especially pore formation in the mitochondrial membrane. Of note, MLKL is a key protein in the execution of programmed necrosis, with its property of translocation across the plasma membrane and forming transmembrane pores. Since Meth treatment significantly decreases MMP and promotes cytochrome c release, it allowed us to assume that MLKL might be involved in the Meth-induced mitochondrial membrane impairment. Therefore, MLKL and its upstream proteins, including RIPK1 and RIPK3, were examined.

Our data suggested that Meth treatment leads to an increase in TNF-α, RIPK1, RIPK3, and MLKL expression, a elevated percentage of PI-positive cells as quantified by flow cytometry (Supplementary Figure S2), LDH release, and overall PI-positive cells, suggesting that Meth plays a specific role in stimulation of neuronal necroptosis.

Mitochondria have long been regarded as the primary regulators of regulated cell death (RCD) [29,45]. Studies have demonstrated that disruption of mitochondrial function and structural integrity associated with RCD closely relates to regulating inflammatory responses essential for organismal homeostasis [46]. For example, the increased TNF-α binds to its receptor TNFR-1, leading to the recruitment of the adaptor TNFR-associated death domain protein (TRADD), which bridges the interaction between TNFR-1 and RIPK1 [47]; the activated RIPK1 then forms protein complexes with RIPK3 and MLKL, and the necroptosome is formed in the current study. Nec-1, one blocker for necroptosome formation [48], significantly attenuated the Meth-induced increase in necroptotic proteins and mitochondrial dysfunction, implying the specific role of the necroptosome in impairing the mitochondria. Although clinical applications of MLKL inhibitors remain limited, elevated systemic levels of RIPK1 and MLKL have been reported in patients with inflammatory conditions such as non-alcoholic fatty liver disease [49], highlighting their pathophysiological relevance. It should be noted, however, that Nec-1 may have off-target effects—including inhibition of IDO-1, an enzyme involved in kynurenine production [50], which can influence immune function and NAD^+^ metabolism, as well as potential inhibition of other kinases. Therefore, the application of Nec-1 in clinical treatment needs to be handled with extreme caution.

Recent studies have shown that p-MLKL translocates to mitochondria and induces a microtubule-dependent release of mitochondrial DNA (mtDNA), which is ascribed to the mitochondrial “membrane leak”, while suppression of MLKL formation restricts this process [51]. This phenomenon is in line with our hypothesis that MLKL might affect mitochondrial integrity. In accordance with our assumption, Meth treatment stimulated the translocation of MLKL to the membrane of the mitochondria, forming “holes” similar to the cell membrane [25]. The assumption was further supported by the evidence that Meth administration can upregulate the expression of the pro-death protein BAX [52], a protein which can recruit MLKL to lysosomes and induce lysosomal membrane permeabilization [53], reflecting the characteristic role of MLKL in membrane permeabilization.

Interestingly, the MLKL–mtROS signaling seems to form a positive feedback loop since the mitochondrial ROS inhibitors also ameliorated mitochondrial dysfunction and the active MLKL-associated necroptosis. The reason for this is unknown; we suspect that mitochondrial ROS contribute to necroptosis by facilitating RIPK1 phosphorylation, culminating in its activation and subsequent necroptosis [54,55,56]. Additionally, RIPK3 kinase triggers the activation of pyruvate dehydrogenase 3 in a feed-forward manner, boosting aerobic respiration and augmenting ROS production [32]. Importantly, Meth treatment markedly induced mitochondrial fragmentation in a Drp-1-dependent manner [57], a process potentially linked to MLKL translocation, as evidenced by studies showing that cadmium exposure triggers MLKL-dependent Drp1 recruitment, resulting in excessive mitochondrial fission and mtROS overproduction [58]. Nonetheless, further evidence is needed to clarify how MLKL pore formation directly interacts with Meth-induced mtROS generation [59,60]. Together, these findings indicate that Meth triggers Drp-1-mediated mitochondrial fragmentation, membrane leakage, loss of membrane potential, and increased ROS production, collectively amplifying neuronal damage through a reinforced cycle of MLKL activation and mtROS-driven necroptotic signaling.

However, the current study also has some potential limitations. First, we can not rule out the effects of Gasdermin-D in Meth-induced mitochondrial membrane impairments since its similar characteristics of “hole-making” in the membrane, and more research is needed in future work. Second, although we used pharmacological inhibition of the RIPK1–RIPK3–MLKL axis, there still may be the possibility of off-target effects for RIPK1–RIPK3; therefore, the siRNA assay will be performed in future work to specifically knockdown RIPK1, RIPK3, and MLKL and illustrate the roles of the RIPK1–RIPK3–MLKL axis in Meth-induced neurotoxicity.

For clinical treatment, the therapeutic potential of targeting MLKL is significant. Small molecule inhibitors that specifically block MLKL translocation to the mitochondrial membrane could be developed. However, the specificity of these inhibitors needs to be carefully evaluated to avoid off-target and side effects.

**Figure 6:**
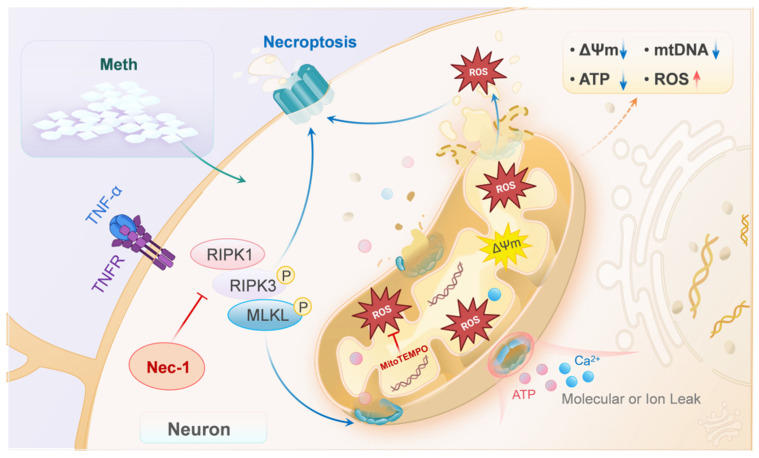
A schematic depicting the role of the MLKL in Meth-induced neuronal necroptosis. With the stimulation of Meth, RIPK1 forms form a complex with RIPK3, leading to phosphorylation of RIPK3 and activating MLKL, leading to mitochondrial membrane permeability, mtDNA and ATP decrease, mitochondrial ROS generation, and finally neuronal necroptosis.

In summary, our data reveal an unrecognized Meth-induced neuronal necroptosis via RIPK1–RIPK3-mediated MLKL translocation to the mitochondrial membrane (Figure 6), which may provide novel insights into the mechanism of Meth-induced neuronal death, and targeting MLKL could thus be considered as a promising strategy for intervention in Meth-induced neurodegenerative diseases.

## 5. Conclusions

Meth induces necroptosis in primary neuronal cells via mitochondrial dysfunction, especially the “hole-making” characteristic of MLKL in the mitochondrial membrane; therefore, targeting the RIPK1–RIPK3–MLKL axis may provide novel therapeutic ways for mitigating Meth-induced neuronal death.

## Data Availability

The datasets used and/or analyzed during the current study are available from the corresponding author or on reasonable request.

## Supplementary Materials

The following supporting information can be downloaded at: https://www.mdpi.com/article/10.3390/toxics13090736/s1, Figure S1: The primary cortical neurons were treated with Meth (900 μM) for 24 h. TNF-α, TNFR1, TNFR2, RIPK1, RIPK3, and MLKL mRNA levels were detected by qRT-PCR normalized to untreated neurons.The primary cortical neurons were treated with Meth (900 μM) for 24 h. TNF-α, TNFR1, TNFR2, RIPK1, RIPK3, and MLKL mRNA levels were detected by qRT-PCR normalized to untreated neurons. Figure S2: The primary cortical neurons (50 neurons/group; n=3) were treated with Meth (900 μM) for 24 h Flow cytogram. Data are expressed as the mean ± SD. Experiments were repeated at least 3 times * *p* <0.05, ** *p* <0.01, *** *p* <0.001, **** *p* <0.0001 compared to the control group.
