# Supplementary material for: Methamphetamine Exposure Induces Neuronal Programmed Necrosis by Permeabilizing Mitochondria via the RIPK1–RIPK3–MLKL Axis
Source: Toxics. 2025 Aug 30;13(9):736. doi: 10.3390/toxics13090736 (PMC12473512; doi:10.3390/toxics13090736)
Supplement: Supplementary file 1 [file toxics-13-00736-s001.zip › toxics-3815802-supplementary.pdf]

Table S1　Primer sequence for RT-PCR

Gene	Forward primer	Reverse primer
TNF-α	5′-CCTCACCCACACCGTCAG-3′	5′- GCAGGTCCCCCTTCTCCA-3′
TNFR1	5′-AGACCCTGCGATGCTGTATG-3′	5′-CGCAGGTTCATGTCGCAAAG-3′
TNFR2	5′-GTGGGTATGAAGCCCAACCA-3′	5′-AGGCCACTAAAGGAAACGGG-3′
Caspase8	5′- GAGGACATACCCAAACTC -3′	5′- ATCACTGAAGGACACGAT-3′
RIPK1	5′-GAGCACCGACCAATTCTG-3′	5′- TTCCACTTTCCAGTCGCTA-3′
RIPK3	5′-CAACCGAGAACTGCCTTT-3′	5′- GCTGCCTGAATGGTGTATT-3′
MLKL	5′-GGTCTGGGGTGCATTGT-3′	5′- GCTGGTCCTTGGTCTATCC-3′
β-actin	5′- GAGAGGGAAATCGTGCGT -3′	5′- GGAGGAAGAGGATGCGG-3′

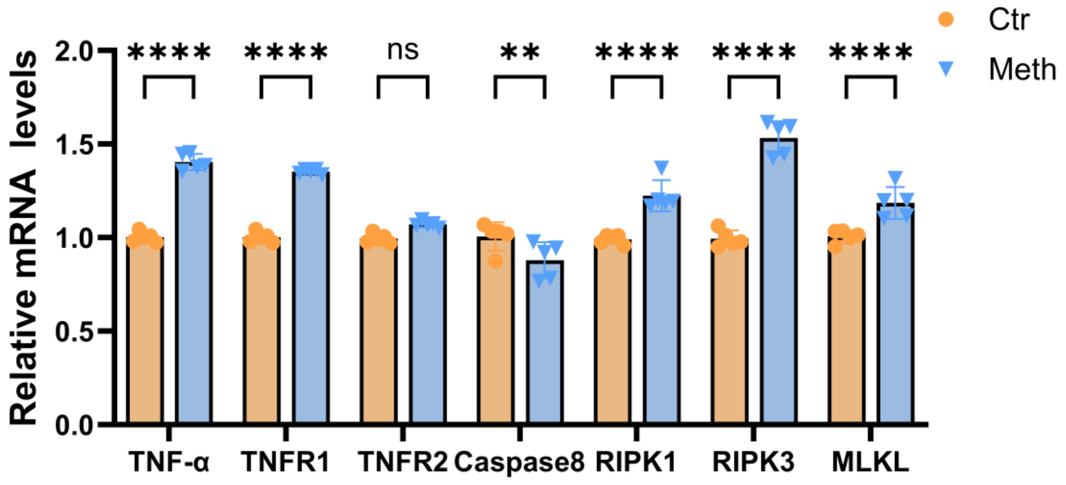

Figure S1: The primary cortical neurons were treated with Meth (900 μM) for 24 h. TNF-α, TNFR1, TNFR2, RIPK1, RIPK3, and MLKL mRNA levels were detected by qRT-PCR normalized to untreated neurons.The primary cortical neurons were treated with Meth (900 μM) for 24 h. TNF-α, TNFR1, TNFR2, RIPK1, RIPK3, and MLKL mRNA levels were detected by qRT-PCR normalized to untreated neurons.

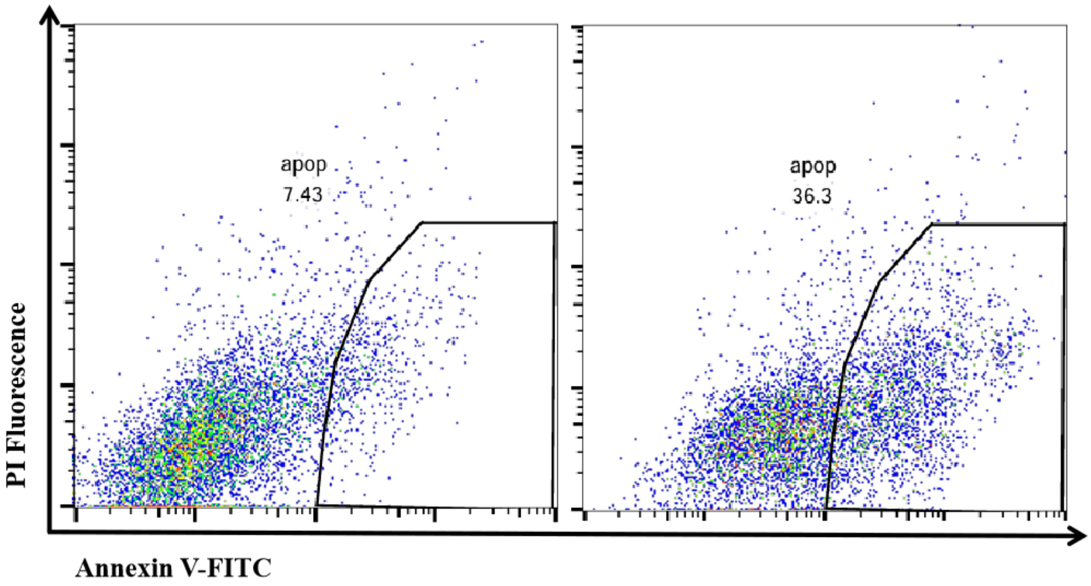

Figure S2: The primary cortical neurons (50 neurons/group; n=3) were treated with Meth (900 μM) for 24 h Flow cytogram. Data are expressed as the mean ± SD. Experiments were repeated at least 3 times *p<0.05, **p<0.01, ***p<0.001, ****p<0.0001 compared to the control group.
